# Accuracy of transient elastography-FibroScan^®^, acoustic radiation force impulse (ARFI) imaging, the enhanced liver fibrosis (ELF) test, APRI, and the FIB-4 index compared with liver biopsy in patients with chronic hepatitis C

**DOI:** 10.6061/clinics/2017(09)01

**Published:** 2017-09

**Authors:** Taisa Grotta Ragazzo, Denise Paranagua-Vezozzo, Fabiana Roberto Lima, Daniel Ferraz de Campos Mazo, Mário Guimarães Pessoa, Claudia Pinto Oliveira, Venancio Avancini Ferreira Alves, Flair José Carrilho

**Affiliations:** I Divisao de Gastroenterologia e Hepatologia Clinica, Departamento de Gastroenterologia, Hospital das Clinicas HCFMUSP, Faculdade de Medicina, Universidade de Sao Paulo, Sao Paulo, SP, BR; II Divisao de Gastroenterologia, Faculdade de Ciencias Medicas, Universidade Estadual de Campinas, Campinas, SP, BR; III Departamento de Patologia, Faculdade de Medicina FMUSP, Universidade de Sao Paulo, Sao Paulo, SP, BR

**Keywords:** Hepatitis C Chronic, Liver Cirrhosis, Elastography, Biomarkers/Blood, Disease Progression, Data Accuracy

## Abstract

**OBJECTIVES:**

Although liver biopsy is the gold standard for determining the degree of liver fibrosis, issues regarding its invasiveness and the small amount of liver tissue evaluated can limit its applicability and interpretation in clinical practice. Non-invasive evaluation methods for liver fibrosis can address some of these limitations. The aim of this study was to evaluate the accuracy of transient elastography-FibroScan^®^, acoustic radiation force impulse (ARFI), enhanced liver fibrosis (ELF), the aspartate aminotransferase-to-platelet ratio index (APRI), and the FIB-4 index compared with liver biopsy in hepatitis C.

**METHODS:**

We evaluated chronic hepatitis C patients who were followed at the Division of Clinical Gastroenterology and Hepatology, Hospital das Clínicas, Department of Gastroenterology of University of São Paulo School of Medicine, São Paulo, Brazil, and who underwent liver biopsy. The accuracy of each method was determined by a receiver operating characteristic (ROC) curve analysis, and fibrosis was classified as significant fibrosis (≥F2), advanced fibrosis (≥F3), or cirrhosis (F4). The Obuchowski method was also used to determine the diagnostic accuracy of each method at the various stages of fibrosis. In total, 107 FibroScan^®^, 51 ARFI, 68 ELF, 106 APRI, and 106 FIB-4 analyses were performed.

**RESULTS:**

A total of 107 patients were included in the study. The areas under the ROC curve (AUROCs) according to fibrosis degree were as follows: significant fibrosis (≥F2): FibroScan^®^: 0.83, FIB-4: 0.76, ELF: 0.70, APRI: 0.69, and ARFI: 0.67; advanced fibrosis (≥F3): FibroScan^®^: 0.85, ELF: 0.82, FIB-4: 0.77, ARFI: 0.74, and APRI: 0.71; and cirrhosis (F4): APRI: 1, FIB-4: 1, FibroScan^®^: 0.99, ARFI: 0.96, and ELF: 0.94. The accuracies of transient elastography, ARFI, ELF, APRI and FIB-4 determined by the Obuchowski method were F0-F1: 0.81, 0.78, 0.44, 0.72 and 0.67, respectively; F1-F2: 0.73, 0.53, 0.62, 0.60, and 0.68, respectively; F2-F3: 0.70, 0.64, 0.77, 0.60, and 0.67, respectively; and F3-F4: 0.98, 0.96, 0.82, 1, and 1, respectively.

**CONCLUSION:**

Transient elastography remained the most effective method for evaluating all degrees of fibrosis. The accuracy of all methodologies was best at F4.

## INTRODUCTION

There are approximately 130 to 150 million carriers of the hepatitis C virus (HCV) worldwide. Approximately 55-85% of patients with HCV develop the chronic form of the disease, and approximately 15-30% of these patients are at risk of developing cirrhosis within 20 years of diagnosis. Approximately 300,000-500,000 people die each year as a result of complications arising from HCV 1. In Brazil, hepatocellular carcinoma (HCC) is one of the most frequent complications of cirrhosis caused by HCV 2,3.

Liver biopsy is still considered the gold standard for liver tissue evaluation, allowing the ascertainment not only of the degree of fibrosis but also of other important parameters, such as inflammation, necrosis, steatosis, and the presence of hepatic iron in the sample obtained 4. However, liver biopsy is an invasive procedure that carries risks, sometimes causing pain, hemorrhage, and even death, among other complications 5. Issues regarding the quality of the liver samples and interpretation of the results can also occur. The quality of a liver biopsy is generally related to the length and number of portal spaces evaluated 6,7. The results of the pathological anatomy can vary according to the subjective interpretation of the individual pathologist 8.

Due to these limitations, non-invasive methods of liver fibrosis evaluation have been studied intensely and have improved over recent decades. These methods can be divided into two categories, namely, indirect markers, which can be assessed by routine clinical exams (e.g., aminotransferases and platelet count) 9, and direct markers, which include serum levels of substances involved in the molecular pathogenesis of fibrosis, such as matrix metalloproteinases, hyaluronic acid, and cytokines [tumor necrosis factor α (TNF-α) and transforming growth factor β (TGF-β)] 10.

Elastography methods use an existing modality, such as ultrasound (US), to observe the internal tissue deformations that occur in response to an applied force and convert the resulting information to a suitable form for display. A wide variety of approaches have evolved, both for applying the force and for measuring and displaying the tissue’s response after applying a force that is either dynamic (e.g., by thumping or vibrating) or that varies so slowly that it is considered "quasi-static" (e.g., by probe palpation). The deformation may be represented in an elasticity image (elastogram) or as a local measurement of tissue displacement that may be detected and displayed directly [Acoustic Radiation Force Impulse (ARFI) imaging]. Another form of representation is through the regional values of their speed (without making images) using methods referred to herein as transient elastography (TE) 11.

The aim of this study was to evaluate the accuracy of TE-FibroScan^®^, ARFI imaging, enhanced liver fibrosis (ELF), the aspartate aminotransferase-to-platelet ratio index (APRI), and FIB-4 compared with liver biopsy in chronic hepatitis C patients.

## PATIENTS AND METHODS

We performed a prospective study evaluating treatment-naive patients chronically infected with HCV, who were on the waiting list for liver biopsy at the outpatient clinic of the Division of Clinical Gastroenterology and Hepatology, Hospital das Clínicas, Department of Gastroenterology of University of São Paulo School of Medicine, São Paulo, Brazil (HC-FMUSP), from August 3, 2012, to May 31, 2014. A total of 107 patients were included, with 107 liver biopsies, 107 TE exams, 106 APRI and FIB-4 exams, 68 ELF exams and 51 ARFI exams performed.

The liver biopsies were performed with a 14-G Tru-Cut™ needle (15cm) (Medical Technology, Gainesville, FL, USA). Liver histology was examined by the same experienced liver pathologist from the Department of Pathology of HC-FMUSP to avoid operator bias in the analysis. The selected samples had a minimum of 5 portal spaces (a mean of 13 portal spaces: 43 biopsies had between 5 and 10 portal spaces and 64 had between 11 and more than 35 portal spaces) and a mean size of 15 mm (varying between 7 and 23 mm). The classification score used for analysis of the biopsies was the METAVIR score 12.

The inclusion criteria were as follows: 1. HCV PCR-RNA positivity for at least 6 months and a clinical or histopathological diagnosis of treatment- naive HCV; 2. negative serological test for hepatitis B or HIV co-infection; and 3. representative liver biopsy (minimum of 5 portal spaces, non-subcapsular fragment) performed 60 days prior to the exams. The exclusion criteria were as follows: 1. refusal to provide informed consent; 2. patient under 18 or over 70 years of age; 3. unavailability of liver biopsy (contraindication); 4. biopsies performed more than 60 days before the evaluation; 5. non-representative liver biopsy; 6. clinical suspicion or image evidence of HCC; 7. ascites; 8. body mass index (BMI) ≥30kg/m^2^ and 9. previous treatment for HCV. 10. unreliable FibroScan^®^ results (as described later).

The following examinations were utilized for fibrosis evaluation: TE-FibroScan^®^, ARFI, ELF, APRI, and FIB-4.

FibroScan^®^ was developed by Echosens (Paris, France) in 2003 13. The equipment consists of a 5-MHz US transducer coupled to a base with a vibratory axis, and it uses VCTE technology (elastography technology with velocity-controlled impulses). The vibration emitted by the transducer is of medium amplitude (2 mm) and low frequency (50 Hz). It produces a wave through the liver tissue, which allows estimation of the elasticity of the hepatic parenchyma through the equation E=3pV^2^, where: E=elasticity, p=density (a constant in tissue), and V=velocity of wave propagation. The denser the tissue, the faster the wave propagates. The results of liver elasticity measurements are expressed in kilopascals (kPa) in an interval of 2.5-75 kPa. The section analyzed corresponds to a cylinder 1 cm in diameter and 4 cm in length, corresponding to a volume 100-fold larger than that obtained with liver biopsy 11,13-15. A liver stiffness assessment is generally considered reliable when the following criteria are fulfilled: 10 valid measurements, success rate >60%, and ratio of the interquartile range to the median (IQR/M) ≤30% 16-18. Patients with invalid/ unreliable measurements were excluded from the study. The TE examinations in this study were performed by the same experienced and highly trained operator, thus eliminating the risk of inter-observer bias. All patients fasted for at least three hours prior to examination because the FibroScan^®^ reading (in kPa) varies immediately following food consumption, altering the result 19. The inclusion criteria of BMI ≤30 kg/m^2^ and skin-liver distance are factors that affect the choice of transducer used. The M transducer was used for all TE examinations in this study to avoid potential bias in interpreting the results in kPa because when both the M, and XL transducers are used, different results can occur 20-22.

ARFI imaging (Siemens Acuson S2000, Virtual Touchä tissue quantification; Siemens, Erlangen, Germany) is another tool for evaluating liver stiffness that can be incorporated into a conventional US machine. Transducers sensitive to the propagation of acoustic pulses of a frequency of 2.67 Hz generate shear waves that propagate in the tissue perpendicular to the direction of the acoustic impulse. The shear waves are then tracked using US based on correlation in a small region of interest (ROI) of 5 mm x 10 mm, which the operator can easily locate in the field of vision up to a depth of 8 cm with a convex catheter. This allows the detection of liver stiffness through the velocity of propagation of the shear wave, which is linearly correlated with the stiffness of the liver 11,23,24. The results are expressed in meters per second (m/s) with an interval scale of 0.5-4.4 m/s and precision of ±20% related to the interval 25. A result is considered high quality when 10 valid measurements are recorded with an IQR/M ratio <30% and a success rate of ≥60% 26. The examination is performed at the height of the medial axillary line and the xiphoid appendage with a projection of S8.

As a direct method utilizing biomarkers in the blood, ELF is a serum test that yields a single value, combining quantitative measures of hyaluronic acid (HA), pro-peptide amino-terminal of pro-collagen type III (PIIINP), and tissue inhibitor metalloproteinase 1 (TIMP-1) in human serum in an algorithm 10,27. The ADVIA Centaur^®^ CP was used for ELF, following formula was applied to yield results: 2.494+0.846 In (C HA) + 0.735 In (C PIIINP) + 0.391 In (C TIMP-1).

Regarding the methods using indirect biomarkers, APRI and FIB-4 were calculated through the following scores:

*APRI score*= [(AST/ULN) 100]/platelet count 10^9^/L. 28

(ULN: upper limit of normal)

*FIB-4 score*= {[age (yr) x AST (U/L)] / [platelet count (10^9^/L) x ALT (U/L)]} 29

**Figure 1:**
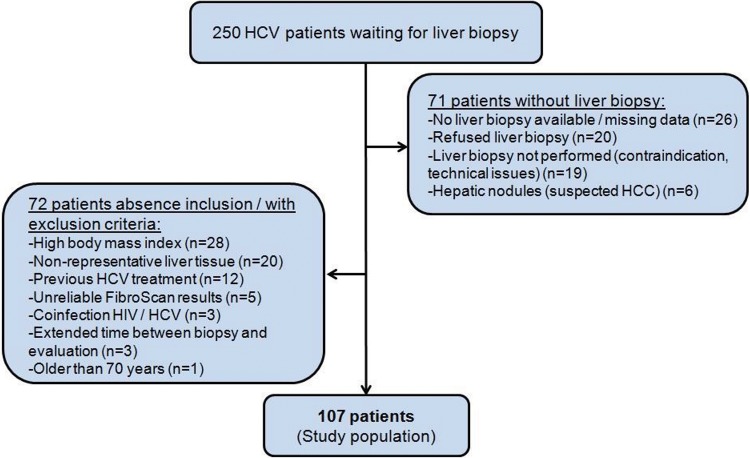
Flowchart of study population enrollment. HCV= hepatitis C virus, HCC= hepatocellular carcinoma.

The study was approved by the Ethics Committee of HC-FMUSP. Of the 250 patients eligible for the study, 143 were excluded for various reasons, as described in Figure 1. Among the patients with liver biopsy, 20 were excluded due to non-representative liver histology (<5 portal spaces and/or non-subcapsular fragment), and 5 patients were excluded because of unreliable FibroScan^®^ results (one patient presented with narrow intercostal space, 3 patients had IQR/M >30% and one patient had an exam success rate <60%). A total of 107 patients were included, as shown in Figure 1.

### Statistical analyses

The R package version 3.2.1(R Core Team, Vienna, Austria) was used for statistical and graph analyses. The performance of the non-invasive methods was estimated using ROC curves (using the package pROC version 1.8) by identifying the optimal cut-off points of different degrees of liver fibrosis in terms of sensitivity and specificity. The area under the ROC curve (AUROC) indicates the accuracy of the studied methods. The Obuchowski method was used to determine the accuracy of the non-invasive methods of liver fibrosis evaluation 30,31

Comparative analyses of more than two groups were performed using analysis of variance (ANOVA), the Levene statistic and the Kruskal-Wallis test. For multiple analyses, or analyses of two groups with more than two other groups, the Tukey and non-parametric Tukey tests were used. For qualitative (categorical) variables, Fisher's exact test and the chi-squared test were used. Statistical significance was defined as *p*<0.05.

## RESULTS

A total of 107 patients were included. The population's gender was 50.4% (n=54) female, with 67.2% (n=72) of white ethnicity and 31.7% (n=34) of black ethnicity. In terms of exposure to HCV, 27.2% (n=29) had a history of blood transfusion and 25.2% (n=27) contracted the virus through tattooing. Genotype 1 was the most common, found in 81.9% of cases. One of the assessed patients had genotype 1 and genotype 2 co-infection. The degree of fibrosis according to the METAVIR scale was as follows: F0=7.4% (n=8), F1=40.1% (n=43), F2=28.9% (n=31), F3=21.5% (n=23), and F4=1.8% (n=2). Table 1 shows anthropometric and laboratory data according to the degree of fibrosis, demonstrating the importance of clinical and laboratory parameters according to liver disease severity.

**Figure 2:**
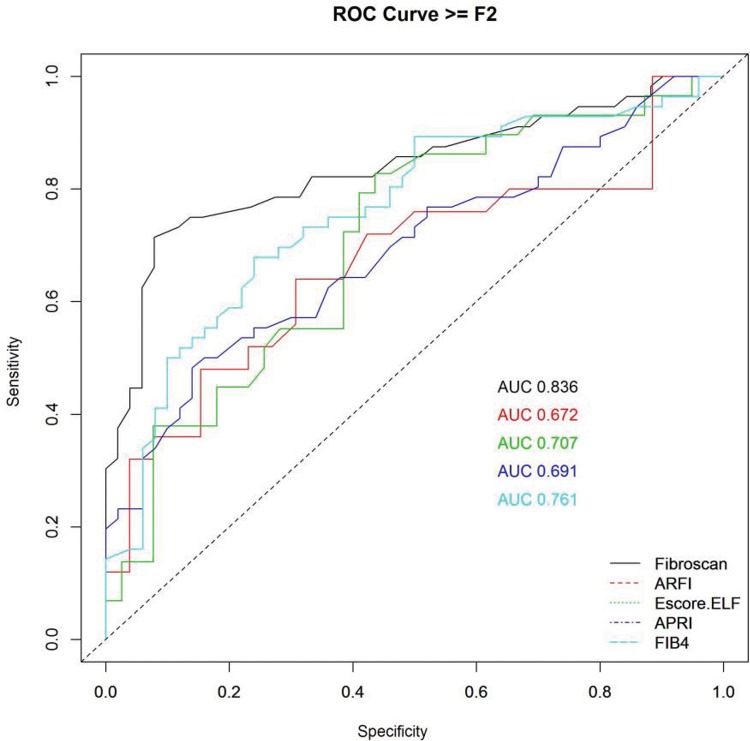
ROC curve for significant fibrosis (≥F2).

**Figure 3:**
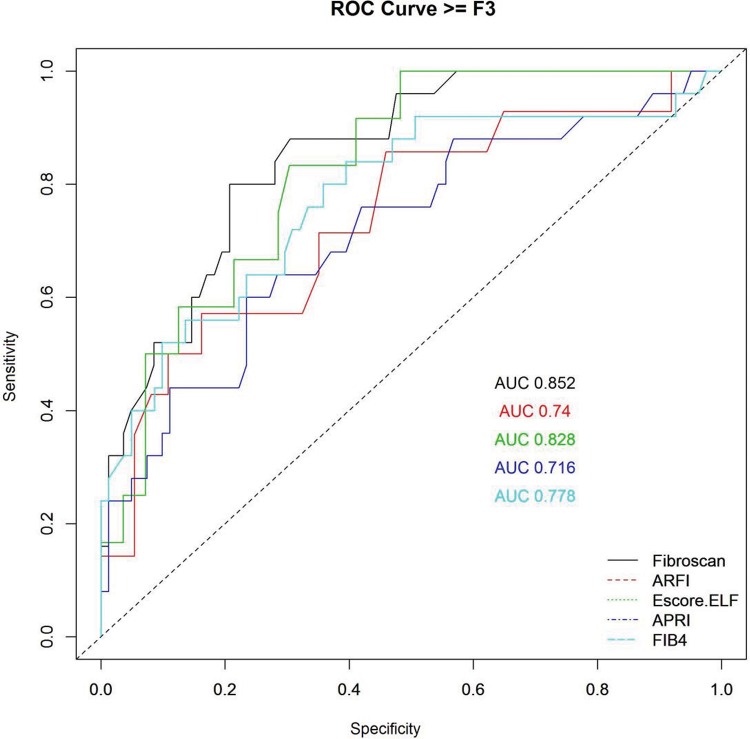
ROC curve for advanced fibrosis (≥F3).

**Figure 4:**
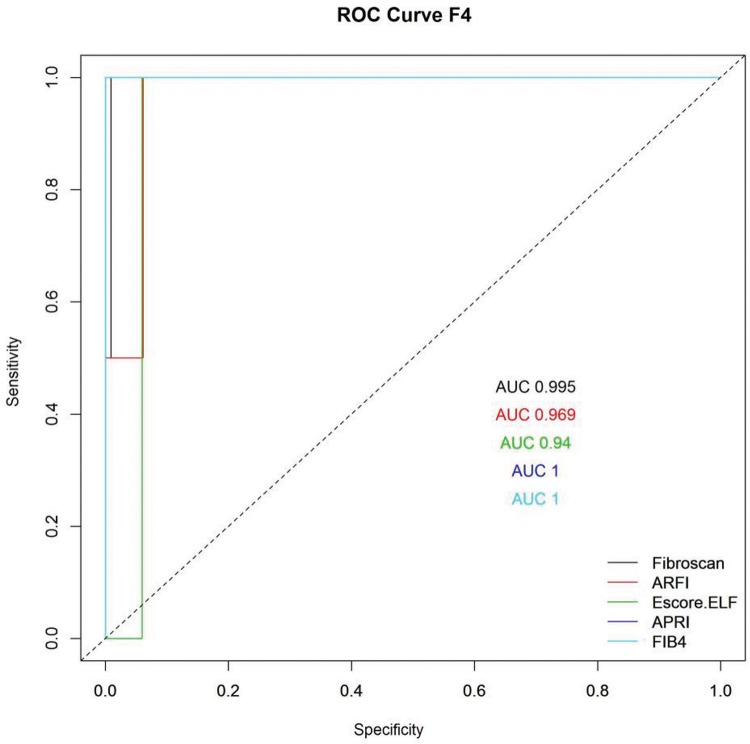
ROC curve for cirrhosis (F4).

Figures 2, 3 and 4 and Tables 2, 3 and 4 present ROC curves and statistical characteristics for significant fibrosis (≥F2), advanced fibrosis (≥F3) and cirrhosis (F4), respectively.

Table 5 shows the accuracy of the non-invasive methods of liver fibrosis evaluation.

## DISCUSSION

Several factors can influence the results of non-invasive methods of liver fibrosis evaluation. Regarding TE, fasting, operator bias, and anthropometric characteristics can influence the success and reliability of FibroScan^®^ 32,33. TE examinations were performed taking into account these variables, as described in the Methods section. Two factors can influence the results of ARFI. High BMI can cause underestimation of fibrosis, and a skin-liver distance >2.5 cm increases the discrepancy compared with liver biopsy 34,35. Gender can also be a confounding factor in ARFI. Male patients typically have higher ARFI values than female patients 23. ELF can be affected by age; the frequencies of cardiovascular disease and chronic inflammatory diseases are higher in older populations 36,37.

With significant fibrosis defined as ≥F2, FibroScan^®^ showed a higher degree of accuracy than the other methods assessed. An AUROC of 0.836 is considered an extremely good result 38. ELF also showed good results, with an AUROC of 0.707. ARFI and APRI both showed sufficiently satisfactory results, with AUROC values of 0.672 and 0.691, respectively. In the ROC curve for APRI, it was not possible to distinguish a cut-off point between F1 and F2; therefore, it was not possible to separate F1/F2 in the analysis of group F2F3F4 (≥F2). FibroScan^®^ and ELF were extremely effective in identifying advanced fibrosis (≥F3), with AUROCs of 0.85 and 0.82, respectively. FIB-4, ARFI, and APRI all yielded results that were considered good, with AUROCs of 0.77, 0.74, and 0.71, respectively. All of the methods were excellent in identifying cirrhosis (F4) with AUROCs of 0.94 for ELF, 0.969 for ARFI, 0.995 for FibroScan^®^, 1 for APRI and 1 for FIB-4.

In this study, FibroScan^®^ was consistently accurate in classifying degrees of fibrosis as ≥F2, ≥F3, or F4 with AUROCs of 0.83, 0.85, and 0.99 respectively, which was consistent with the findings of Castera et al. 16 (≥F2 AUROC 0.83, Se: 67, Sp: 89; ≥F3 AUROC: 0.90, Se: 73, Sp: 91; and F4 AUROC: 0.95, Se: 87, Sp: 91) and of Ziol et al. 39 (≥F2 AUROC: 0.79, Se: 56, Sp: 91; ≥F3 AUROC: 0.96, Se: 86, Sp: 85; F4 AUROC: 0.97, Se: 86, Sp: 96). The specificity and sensitivity of FibroScan^®^ were highest at ≥F4 40. FibroScan^®^ has also proven to be an effective method of assessing fibrosis progression for a variety of other pathologies, such as hepatic steatosis 41. It is also applicable in the evaluation of fibrosis progression and complications, such as portal hypertension 42,43, and in the clinical treatment of HCV and other chronic liver diseases 44,45.

Gara et al. 46 showed that, despite the high sensitivity and specificity of FibroScan^®^ and ARFI in the diagnosis of cirrhosis, due to the possibility of false-positives, it is always necessary to view the results in the context of clinical exams or an imaging exam. ARFI had AUROCs of 0.67 for ≥F2 (Se: 64, Sp: 69), 0.74 for ≥F3 (Se: 57, Sp: 74), and 0.97 for F4 (Se: 100, Sp: 97) in this study. ARFI showed less accuracy than that found in the literature. In the meta-analysis by Friedrich-Rust et al. 21, the mean AUROCs for ARFI were 0.87, 0.91, and 0.93 for ≥F2, ≥F3 and F4, respectively. A meta-analysis by Nierhoff et al. 47 yielded AUROCs of 0.84, 0.89 and 0.91 for ≥F2, ≥F3, and F4 respectively, indicating that FibroScan^®^ is a good tool for diagnosing significant fibrosis, and an excellent tool for diagnosing advanced fibrosis or cirrhosis. Regarding the findings related to ARFI and FibroScan^®^, this study revealed differences from the meta-analysis of Bota et al. 48, who found FibroScan^®^ and ARFI to be equally accurate in diagnosing significant fibrosis and cirrhosis. Our study revealed FibroScan^®^ to be superior to ARFI in diagnosing significant fibrosis. Perhaps the predominance in this study of fibrosis at stages F0, F1, and F2 could account for this divergence from the literature. However, there are studies that found equivalency between FibroScan^®^ and ARFI for diagnosing advanced fibrosis and cirrhosis but that revealed FibroScan^®^ as the better choice for identifying significant fibrosis 49. In two comparative analyses 50,51, both ARFI and APRI demonstrated the ability to evaluate the progression of advanced fibrosis and cirrhosis in HCV, with ARFI exhibiting slightly better accuracy 50,51.

For ELF, we found AUROCs of 0.71 for ≥F2 fibrosis (Se: 82, Sp: 56), 0.82 for ≥F3 fibrosis (Se: 83, Sp: 69), and 0.94 for F4 fibrosis (Se: 100, Sp: 100). Despite presenting inferior accuracy for ≥F2 fibrosis compared with other studies 10,52, ELF produced results similar to those of Parkes et al.^53^, with AUROCs of 0.74, 0.84 and 0.90 for ≥F2, ≥F3, and F4 fibrosis, respectively, in a specific HCV population. Fagan et al. 54 found that a cut-off score for ELF of ≥9.8 had a sensitivity of 74.4% and a specificity of 92.4% for advanced fibrosis. In this study, the cut-off for ≥F3 fibrosis was 9.4 with Se=83 and Sp=69.

In the present study, APRI had a ≥F3 AUROC: 0.71 and an F4 AUROC of 1. This result differ from the original work, which revealed a ≥F3 AUROC of 0.88 and an F4 AUROC of 0.94 24. APRI could not identify the individual stages of fibrosis, and the fibrosis of some patients remained unclassified when the initial cut-off was applied. Furthermore, the appropriate definition of the limits of normal AST remains uncertain. Each laboratory establishes a different value for the upper limit of normal 55. APRI and FIB-4 are excellent for cirrhosis, but care should be taken with regard to the few patients who have test results close to normal levels. These patients are at risk for false-negative results; therefore, the tests should be performed alongside imaging examinations 56.

Among the various comparative studies of non-invasive methods of liver fibrosis assessment, Crisan et al. 57 showed good results for the accuracy of APRI, FIB-4, and FibroScan^®^ in diagnosing ≥F3 fibrosis. Peterson et al. 58 evaluated APRI and ELF in case of significant fibrosis. That study differed from the others in that it did not use the METAVIR classification, but it demonstrated that one methodology can complement the others in some cases. Poynard et al. 59 conducted a meta-analysis of a variety of chronic liver diseases and confirmed the accuracy of FIB-4 and APRI in identifying advanced fibrosis and cirrhosis in HCV.

The results using the Obuchowski method revealed the degree of fibrosis, and it was impossible to distinguish between significant fibrosis, advanced fibrosis, and cirrhosis. In almost all stages of fibrosis, FibroScan^®^ was the most accurate method. It was most accurate at the extremes of F1 and F4, with values of 0.81 and 0.98, respectively, and demonstrated lower accuracy at intermediate stages. ELF showed exactly the same accuracy at F4 in this study as the only other published study that used the Obuchowski method 60.

A limitation of our study is the small sample size, especially in patients at the extremes of the classification of hepatic fibrosis, F0 and F4. This may have had an impact on the results, as previously discussed.

FibroScan^®^ was the most accurate method in diagnosing significant fibrosis (≥F2) and advanced fibrosis (≥F3) (with AUROCs of 0.84 and 0.85 respectively). APRI and FIB-4 were also very accurate in identifying cirrhosis. The accuracy of all the methodologies was best at F4, but the TE remained the most effective method for evaluating all degrees of fibrosis.

## AUTHOR CONTRIBUTIONS

Ragazzo TG designed the study, analyzed the data and helped drafting the manuscript. Paranagua-Vezozzo D designed the study and helped drafting the manuscript. Lima FR analyzed the data and helped drafting the manuscript. Mazo DF and Pessoa MG designed the study and helped drafting the manuscript. Oliveira CP designed the study and analyzed the data. Alves VA and Carrilho FJ designed the study, analyzed the data and helped drafting the manuscript.

## Figures and Tables

**Table 1:** Correlation of anthropometric and laboratory data with fibrosis stage according to METAVIR score (means±standard deviation).

	Degree of Fibrosis				
	F0(n=8)	F1(n=43)	F2(n=31)	F3 (n=23)	F4(n=2)
Age (years)	40.6±9.8	44.8±10.9	49.9±9.9	53.8±8.9	56.5±3.5
BMI	25.5±2.7	24.7±2.8	24.9±3.0	25.6±2.6	23.9±5.5
WC (cm)	94.3±8.1	92.1±6.7	92.1±8.3	94.5±7.8	92±15.5
SLD (cm)	1.6±0.1	1.5±0.2	1.5±0.3	1.6±0.4	1.6±0.7
AST (U/L)	25.8±7	35.9±13.2	46.5±31.3	57.2±39.7	181±15.5
ALT (U/L)	32.1±16.3	47.8±24.4	65.9±67.7	75.0±57.6	136.5±16.2
AP (U/L)	66.7±16.1	66.3±17.9	71.7±22.1	71.2±23.3	227.5±136.4
γGT(U/L)	56.1±69.8	54.4±41.6	142.2±169.3	105.4±95.9	249±16.9
ALB (g/dL)	4.2±0.2	4.4±0.3	4.5±0.2	4.3±0.4	4.05±0.2
INR	1.0±0.1	1.0±0.1	1.0±0.1	1.0±0.1	1.1±0.1
PTL (/mm^3^)	237x103±36x103	234x103±57x103	214x103±46x103	194x103±66x103	118x103±35x103

BMI=body mass index, WC=waist circumference, SLD=skin-liver distance, AST=aspartate aminotransferase, ALT=alanine aminotransferase, FA=alkaline phosphatase, γGT=gamma glutamyltransferase, ALB=albumin; INR=international normalized ratio, PTL= platelet count.

**Table 2:** Statistical characteristics of significant fibrosis (≥F2).

	FibroScan^®^		ARFI		ELF		APRI		FIB4	
	Estimate	95% CI	Estimate	95% CI	Estimate	95% CI	Estimate	95% CI	Estimate	95% CI
cut-off	6.5 kPa		1.22m/s2		8.98		0.67		1.29	
Se	0.71	0.58 - 0.83	0.64	0.43 - 0.82	0.83	0.64 - 0.94	0.48	0.35 - 0.62	0.68	0.54 - 0.8
Sp	0.92	0.81 - 0.98	0.69	0.48 - 0.86	0.56	0.4 - 0.72	0.86	0.73 - 0.94	0.76	0.62 - 0.87
PPV	0.91	0.79 - 0.95	0.67	0.45 - 0.84	0.59	0.42 - 0.83	0.79	0.63 - 0.87	0.76	0.62 - 0.85
NPV	0.75	0.62 - 0.92	0.67	0.45 - 0.84	0.81	0.62 - 0.9	0.6	0.46 - 0.8	0.68	0.54 - 0.82
dlr.positive	9.11	3.5 - 23.67	2.08	1.09 - 3.97	1.9	1.28 - 2.81	3.44	1.65 - 7.21	2.83	1.67 - 4.78
dlr.negative	0.31	0.2 - 0.47	0.52	0.29 - 0.93	0.31	0.13 - 0.71	0.6	0.46 - 0.79	0.42	0.28 - 0.64
AUROC	0.83	0.75 - 0.91	0.67	0.51 - 0.82	0.70	0.58 - 0.83	0.69	0.59 - 0.79	0.76	0.66 - 0.85

Se=sensitivity, Sp=specificity, PPV=positive predictive value, NPV=negative predictive value, dlr.positive=likelihood ratio positive, dlr.negative=likelihood ratio negative, AUROC=area under the ROC curve.

**Table 3:** Statistical characteristics of advanced fibrosis (≥F3).

	FibroScan^®^		ARFI		ELF		APRI		FIB4	
	Estimate	95% CI	Estimate	95% CI	Estimate	95% CI	Estimate	95% CI	Estimate	95% CI
cut-off	7.1kPa		1.41m/s2		9.47		0.67		1.22	
Se	0.8	0.59 - 0.93	0.57	0.29 - 0.82	0.83	0.52 - 0.98	0.6	0.39 - 0.79	0.84	0.64 - 0.95
Sp	0.79	0.69 - 0.87	0.84	0.68 - 0.94	0.7	0.56 - 0.81	0.77	0.66 - 0.85	0.6	0.49 - 0.71
PPV	0.54	0.41 - 0.8	0.57	0.35 - 0.82	0.37	0.25 - 0.85	0.44	0.32 - 0.66	0.4	0.29 - 0.72
NPV	0.93	0.83 - 0.96	0.84	0.61 - 0.94	0.95	0.81 - 0.97	0.86	0.72 - 0.92	0.92	0.81 - 0.95
dlr.positive	3.86	2.42 - 6.15	3.52	1.49 - 8.34	2.75	1.71 - 4.39	2.56	1.54 - 4.25	2.13	1.55 - 2.93
dlr.negative	0.25	0.11 - 0.56	0.51	0.27 - 0.95	0.24	0.07 - 0.86	0.52	0.32 - 0.86	0.26	0.11 - 0.66
AUROC	0.85	0.77 - 0.92	0.74	0.57 - 0.90	0.82	0.71 - 0.93	0.71	0.59 - 0.83	0.77	0.66 - 0.89

Se=sensitivity, Sp=specificity, PPV=positive predictive value, NPV=negative predictive value, dlr.positive=likelihood ratio positive, dlr.negative=likelihood ratio negative, AUROC area under the ROC curve.

**Table 4:** Statistical characteristics of cirrhosis (F4).

	FibroScan^®^		ARFI		ELF		APRI		FIB4	
	Estimate	95% CI	Estimate	95% CI	Estimate	95% CI	Estimate	95% CI	Estimate	95% CI
cut-off	27kPa		2.37m/s2		11		4.3		6.51	
Se	1	0.16 - 1	1	0.16 - 1	0.2	0.25 - 0.64	1	0.16 - 1	1	0.16 - 1
Sp	0.99	0.95 - 1	0.94	0.83 - 0.99	1	0.93 - 1	1	0.97 - 1	1	0.97 - 1
PPV	0.67	0.26 - 1	0.4	0.18 - 1	1	0.17 - 1	1	0.35 - 1	1	0.35 - 1
NPV	1	0.91 - 1	1	0.81 - 1	0.94	0.85 - 0.98	1	0.91 - 1	1	0.91 - 1
dlr.positive	105	14.93 - 738.46	16.33	5.46 - 48.89	Inf	-	I-	0 - -	Inf	-
dlr.negative	0	0 - NaN	0	0 - 1	0.8	0.80 - 1	0	0 - 1	0	0 - f
AUROC	0.99	0.98 - 1	0.96	0.90 - 1	0.94	0.91 - 1	1	1 - 1	1	1 - 1

Se=sensitivity, Sp=specificity, PPV=positive predictive value, NPV=negative predictive value, dlr.positive=likelihood ratio positive, dlr.negative=likelihood ratio negative, AUROC=area under the ROC curve, NaN=Null Inf=infinity.

**Table 5:** Accuracy according to sequential pairs using the Obuchowski method.

Biopsy	FibroScan^®^	ARFI	ELF	APRI	FIB4
F0 *vs* F1	0.81	0.78	0.44	0.72	0.67
F1 *vs* F2	0.73	0.53	0.62	0.60	0.68
F2 *vs* F3	0.70	0.64	0.77	0.60	0.67
F3 *vs* F4	0.98	0.96	0.82	1	1
